# Inhibition of Autophagy Increases Cell Death in HeLa Cells through Usnic Acid Isolated from Lichens

**DOI:** 10.3390/plants12030519

**Published:** 2023-01-23

**Authors:** Madhuree Kumari, Siya Kamat, Sandeep Kumar Singh, Ajay Kumar, C. Jayabaskaran

**Affiliations:** 1 Department of Biochemistry, Indian Institute of Science, Bangalore 560012, India; 2 Division of Microbiology, Indian Agricultural Research Institute, Pusa, New Delhi 110012, India; 3 Centre of Advanced Study in Botany, Banaras Hindu University, Varanasi 221005, India

**Keywords:** Western Ghats, macrolichens, autophagy, apoptosis, cytotoxicity, usnic acid (UA), HeLa, secondary metabolites

## Abstract

The Western Ghats, India, is a hotspot for lichen diversity. However, the pharmacological importance of lichen-associated metabolites remains untapped. This study aimed to evaluate the cytotoxic potential of lichens of this region. For this, sixteen macrolichens were collected and identified from two locations in the Western Ghats. The acetone extract of *Usnea cornuta* (UC2A) showed significant cytotoxicity towards multiple human cancer cell lines. Interestingly, co-treatment with chloroquine (CQ), an autophagy inhibitor, increased the cytotoxic potential of the UC2A extract. A gas chromatography mass spectrometry (GCMS) study revealed usnic acid (UA), atraric acid and barbatic acid as the dominant cytotoxic compounds in the UC2A extract. Further, UA was purified and identified from the UC2A extract and evaluated for cytotoxicity in HeLa cells. The monodansyl cadaverine and mitotracker red double staining revealed the autophagy-inducing activities of UA, and the inhibition of autophagy was confirmed via CQ treatment. Autophagy inhibition increased the cytotoxicity of UA by 12–16% in a concentration-dependent manner. It also increased lipid peroxidation, ROS levels and mitochondrial depolarization and decreased glutathione availability. A decrease in zeta potential and a 40% increase in caspase 3/7 activity were also noted after CQ treatment of UA-treated cells. Thus, cytotoxicity of UA can be increased by inhibiting autophagy.

## 1. Introduction

One of the leading causes of mortality and morbidity for women worldwide, particularly in India, is human cervical cancer [1]. It is the fourth most commonly reported cancer among women in the world. According to the World Health Organization (WHO), with current measures for the treatment of cervical carcinoma, the number of deaths could increase by half in the coming years [2]. With increasing resistance to the current drugs available against cervical carcinoma, natural products have gained momentum, owing to the rich diversity of secondary metabolites produced by the vast array of flora and fauna, many of which are still unexplored [3,4].

Lichens comprise the symbiotic association between mycobiont(s) and photobiont(s), which can thrive in any climatic conditions. Their synergistic growth and survival have resulted in the production of numerous secondary metabolites, many of which are novel [5,6]. Many lichens have been used in Chinese and Indian traditional practices, due to their cytotoxic, antibacterial, antidiabetic and anti-inflammatory potential [7,8]. The Indian subcontinent harbors a rich diversity of lichens with more than 2450 species, especially in Himalayan and Western Ghats regions [9], and it has been estimated that it covers around 45% of the total lichen diversity of the country [10], though the pharmacological importance of the lichens residing in this region remains neglected.

The Western Ghats region is particularly rich in macrolichens including *Usnea*, *Parmelia* and *Cladonia* sp. [11]. Usnic acid (UA) is a unique metabolite reported in various lichen species or some genera, especially belonging to family *Parmeliaceae.* Previous published report showed numerous biological activities of usnic acid [12,13,14]. Generally, in lichens, usnic acid is present in the two enantiomeric forms, (−)-usnic acid and (+)-usnic acid, which protects the lichen from UV radiation. However, (+)-usnic acid (UA) is well known for inducing cytotoxic activities at low concentrations by inducing apoptotic cell death in human breast cancer, human cervical carcinoma and colon adenocarcinoma [13].

Autophagy is one of the cell death mechanisms whereby the existing cellular materials are delivered to lysosomes for the generation of energy via the degradation of intracellular protein and organelles [15]. Many natural products use autophagy as a mechanism to either protect the cells or destroy them. In terms of cancer death regulation, it shows inhibitory effects on tumor cell growth [16]. Many natural products, including berberine, curcumin, honokiol and ursolic acid, are known to induce tumor-suppressing autophagy via JNK, LC3- II/III, mTOR, MAPK, CD 147 and ROS dependent pathways [17]. On the other hand, the inhibition of autophagy by certain natural products, such as artemisinin, paclitaxel and resveratrol, increases the apoptosis-mediated cell death by modulating Wnt/β-catenin, LC3- III and caspase 3 pathways [18]. Recent studies have shown the apoptosis- and autophagy-inducing activities of UA in hepatocellular carcinoma and gastric carcinoma, though their cell-death inducing or preventing roles are still not deciphered [19,20,21]. Chen et al. [19] found that autophagy induction and JNK activation prevent apoptosis in hepatic cell carcinoma mediated by UA, although the precise balance between cell death and cell survival induced by UA remains to be determined.

This study, for the first time, reports the isolation of UA from lichens of the Western Ghats and its cytotoxic potential against human cervical carcinoma (HeLa) cells. Further, the interplay between autophagy and apoptosis in cell death induction mediated by UA in HeLa cells was also investigated.

## 2. Results and Discussion

### 2.1. Isolation and Characterization of Lichens for Cytotoxic Activities

In total, 16 macrolichens were isolated and identified from the two locations of the Western Ghats, India, in which the majority of the species belonged to the Parmeliaceae family (Table S1). In total, 14 lichens (87.5%) were corticolous, while the remaining lichens were saxicolous. Out of 16 lichens, four belonged to Parmelia sp. and two belonged to Usnea, Collema and Cladonia sp. respectively. Similar diversity of macrolichens was obtained by Balaji and Hariharan [11] from the Bolampatti II Forest Range (Siruvani Hills), Western Ghats. The Western Ghats, India is among the eight hotspots of biodiversity worldwide, which host a diverse range of lichens, though their pharmaceutical importance is not discovered yet.

**Figure 1:**
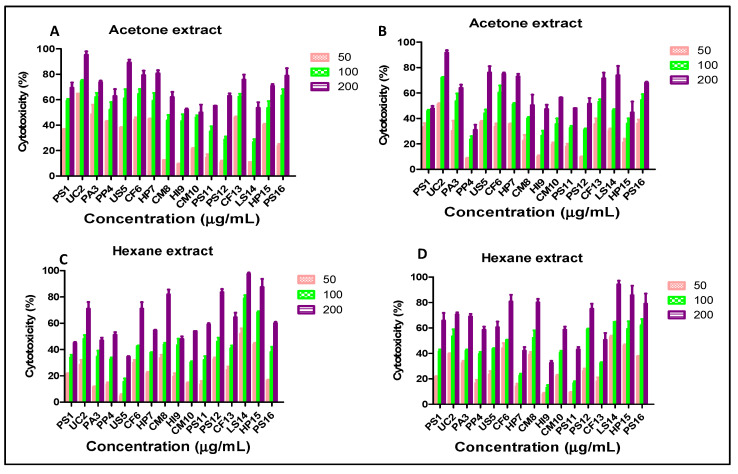
Cytotoxicity of lichen extracts observed via MTT assays after 24 h of treatment; (**A**) acetone extract against HeLa cells; (**B**) acetone extract against MCF-7 cells; (**C**) hexane extract against HeLa cells; (**D**) hexane extract against MCF-7 cells.

The acetone extracts of UC2, PA3, CF6 and CF13 showed good cytotoxicity towards HeLa and MCF-7 cell lines, with UC2 showing the highest cytotoxicity towards HeLa cells (IC_50_, 38.2 ± 1.25 µg/mL) (Figure 1A,B; Table 1). The hexane extracts of CF6, CM8, LS14 and HP15 exhibited moderate cytotoxicity towards HeLa and MCF-7 cells in comparison to that with the acetone extract (Figure 1C,D; Table 1). The results clearly demonstrated the presence of both polar and non-polar cytotoxic compounds in the screened lichen extracts. Though the polar nature of cytotoxic compounds was present in most of the screened lichens, non-polar extracts of CF6, CM8, LS14 and HP15 also need to be screened further for the isolation of non-polar active ingredients.

**Figure 2:**
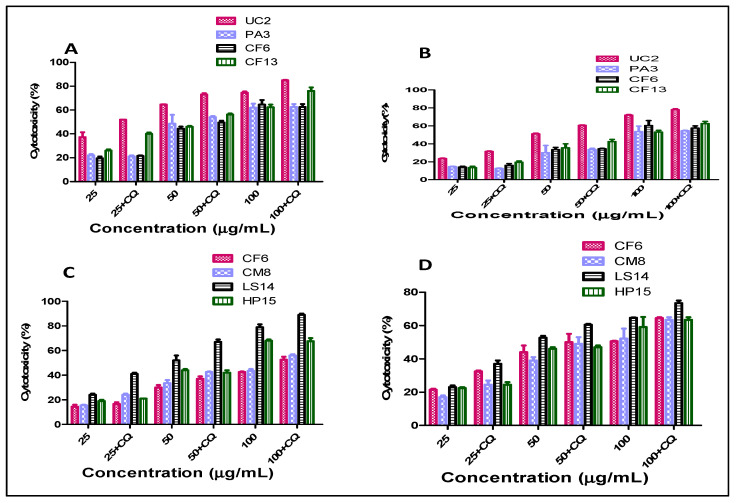
Cytotoxicity of selected lichen extracts in the presence of an autophagy inhibitor, chloroquine (CQ), observed via MTT assays after 24 h of treatment; (**A**) selected lichen acetone extract against HeLa cells (+/−CQ); (**B**) selected lichen acetone extract against MCF-7 cells (+/−CQ); (**C**) selected lichen hexane extract against HeLa cells (+/−CQ); (**D**) selected lichen hexane extract against MCF-7 cells (+/−CQ). Values are the means ± SDs of three independent experiments.

Further, to assess the role of autophagy in the cytotoxicity of the lichen crude extracts, the selected lichen extracts were added with chloroquine, an autophagy inhibitor. A decrease in the IC_50_ of the acetone extract of UC2 and LS14 extracts was observed after the addition of CQ (Figure 2; Table 2). Similarly, the hexane extract of LS14 exhibited a decrease in its IC_50_ value from 41.1 ± 3 to 34 ± 1 µg/mL and 53.7 ± 5 to 47.4 ± 4 µg/mL with HeLa and MCF-7 cells, respectively (Table 2). The highest cytotoxicity was observed with the acetone extract of UC2 against the HeLa cell line after the addition of CQ (IC_50_, 26.01 ± 1 µg/mL). Many of the lichen extracts are known to induce cell death in cancer cells via apoptosis, though the interplay of autophagy in the modulation of cell death has not been deciphered [22,23]. *Usnea intermedia* and *Usnea barbata* extracts demonstrated significant cytotoxicity toward cancer cells by inducing apoptosis [24,25]. Zugic et al. [26] have reported autophagy induction in B16 mouse melanoma and C6 rat glioma using a CO_2_ supercritical extract of Old Man’s Beard (*Usnea barbata*),though the death-inducing or suppressing role of autophagy remains undeciphered [26].

### 2.2. Chemical Composition of UC2A

To determine the cytotoxic metabolites present in UC2A, GCMS was carried out. A total of 29 secondary metabolites matched the hits in the database. The analysis revealed the presence of depsides, phenols and alkaloids in the UC2A extract (Table 3). +(−) Usnic acid, benzoic acid, 2,4-dihydroxy-3,6-dimethyl-, methyl ester (atraric acid) and benzoic acid, 2-hydroxy-4-((2-hydroxy-4-methoxy-3,6-dimethylbenzoyl)oxy)-3,6-dimethyl- (barbatic acid)-methyl ester detected in the GCMS are some of the known cytotoxic secondary metabolites unique to the lichens (Figure S2). The +(−) usnic acid present in abundance (13.3%) in UC2A is known to induce cytotoxicity in multiple cancer cell lines [14]. Atraric acid isolated from lichens, including *Physcia* and *Parmelia* sp., has shown cytotoxic potential against HepG2, A549 and HL-60 cells [27]. Barbatic acid is also a unique metabolite of *Usnea* sp. that can induce cancer cell death [24]. The UC2A extract also showed the presence of octadecanoic and hexadecanoic acid. Interestingly, a vinca alkaloid derivative, apovincamine, was detected in small quantities in the extract of UC2A (0.016%), which has previously shown cytotoxic activities against B16 mouse melanoma cells [28].

### 2.3. Purification and Identification of UA from UC2A Extract

The results of the KCP spot test of UC2 and GCMS analysis of the UC2A extract revealed the presence of UA as a prominent cytotoxic secondary metabolite in the extract; hence, the studies were further carried out for the purification of UA from the UC2A extract.

#### 2.3.1. Thin Layer Chromatography (TLC)

**Figure 3:**
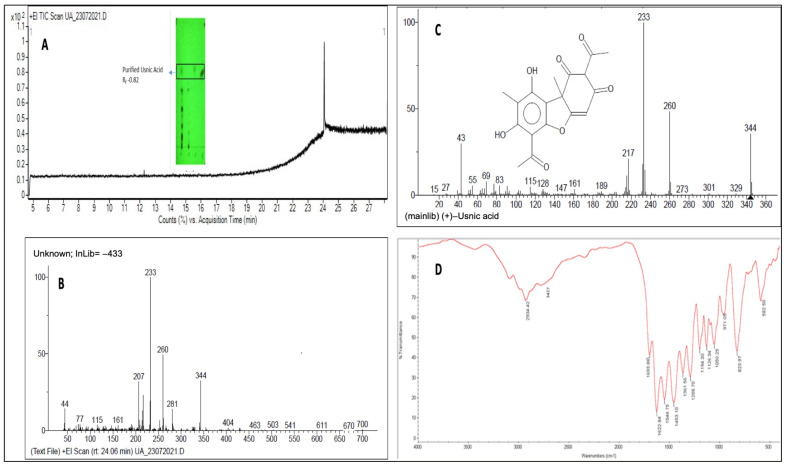
Purification and characterization of usnic acid from *Usnea cornuta* acetone extract (UC2A). (**A**) Inset: TLC analysis of UC2A and the purified compound, usnic acid, with R_f_ = 0.82. Full TIC scan of purified compound, usnic acid, via GCMS at a retention time of 24.06 min. (**B**) +EI scan of peak at 24.06 min of usnic acid in UC2A and (**C**) usnic acid in the NIST database; both fragmentation patterns matched, indicating that the TLC-eluted compound was usnic acid. (**D**) FTIR spectrum of usnic acid from UC2A indicating the presence of functional groups.

After optimization of the solvent systems, the best resolution was observed with the solvent system (toluene:acetic acid, 9:1) for the UC2A extract, when visualized under UV 254 nm. The R_f_ value of the standard UA band and the corresponding band of UC2A extract was found to be 0.82 (Figure 3A-inset). Interestingly, US5 isolated from the other *Usnea* sp. also showed a faint band with an R_f_ value of 0.82. Further, preparative TLC was carried out to obtain a higher quantity of the desired band from the UC2A extract. The obtained yellow colored powder was weighed and dissolved in acetone for further characterization.

#### 2.3.2. GCMS

The GC analyses of the eluted spot indicated a single peak at a retention time 24.06 min (Figure 3A). The MS of the compound indicated abundant fragments of 233, 260 and 344 *m*/*z* (Figure 3B). The NIST database produced a matching hit with ~99 % probability with (+)-usnic acid. This MS of usnic acid (Figure 3C) also indicated abundant fragments of 233, 260 and 344 *m*/*z*, thus confirming the identity of the TLC-eluted spot.

#### 2.3.3. FTIR

The spectrum of FTIR spectroscopy of the UC2A extract (Figure 3D) matched with the peaks that have been reported earlier after FTIR spectroscopy of UA [29,30]. A wide low-intensity peak was observed at 3427 cm^−1^ indicating the stretching vibrations of phenol OH groups of UA. The C-H stretching vibration of the UA aromatic part was noted at 2934 cm^−1^, while C=O stretching of the strong carbonyl non-chelated aromatic ketone was observed at 1693 cm^−1^. A band at 1544 cm^−1^ indicated the conjugated chelated carbonyl, which was also reported by Karabacak et al. [30] in a UA spectrograph. The band at 1453 cm^−1^ corresponded to the C-H bending of alkane, while the band at 971 cm^−1^ depicted the C=C bending of alkene in UA. The presence of a phenolic group in UA was confirmed based on the O-H bending of alcohol at 1361 cm^−1^, while the C-O stretching of alcohol was observed at 1050, 1126 and 1194 cm^−1^. The C=O deformation vibration of the aromatic structure in UA was confirmed based on a high-intensity band at 823 cm^−1^.

### 2.4. Usnic Acid (UA) Isolated from UC2A Extract Shows Concentration-Dependent Cytotoxicity against Cancer Cell Lines

**Figure 4:**
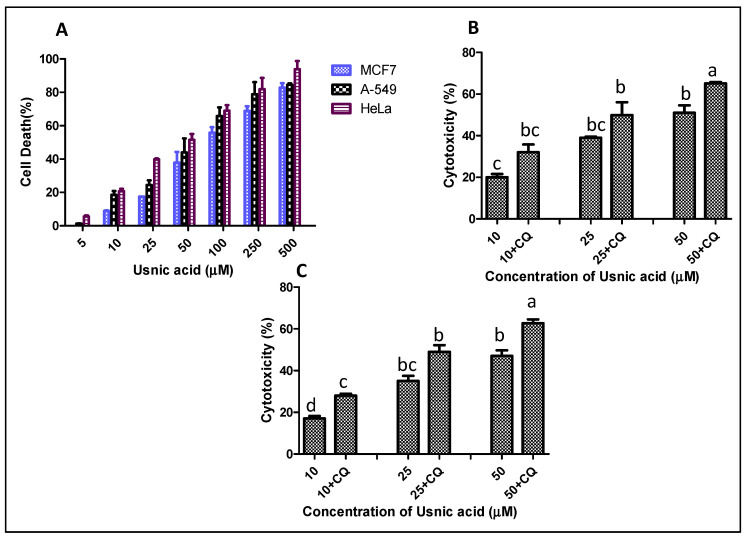
Concentration-dependent cytotoxicity of usnic acid against cancer cell lines after 24 h of treatment. (**A**) Cytotoxicity of usnic acid against MCF-7, A-549 and HeLa cancer cell lines. (**B**) Cytotoxicity of usnic acid in the presence of chloroquine (+/−CQ) via an MTT assay. (**C**) Cytotoxicity of usnic acid in the presence of chloroquine (+/−CQ) via a resazurin assay. Values are the means ± SD of three independent experiments. Means sharing different letters differ significantly from each other at *p* ≤ 0.05.

After isolation, identification and structural elucidation of UA from the UC2A extract, the cytotoxic activities were evaluated against the cancer cell lines MCF7, A-549 and HeLa (Figure 4A).The results showed concentration-dependent cell death in all three cancer cell lines with UA treatment. The lowest IC_50_ was observed with the HeLa cell line (48.65 µM), followed by that with A-549 (84.21 µM) and MCF7 (89.09 µM) cells. Earlier research has shown the UA induces cytotoxicity in HeLa cells with IC_50_ values ranging from 48.5 to 199.2 μM after 72 h of incubation [5,14].

Chloroquine treatment inhibits autophagy by decreasing the degradation of autophagosomes and decreases mitochondrial membrane potential. Autophagy is often linked with apoptosis, although the latter is the most common cell death pathway invoked by anticancer compounds. Autophagy, being a pro-survival cell strategy, diminishes the cytotoxic effect of the treatment. Hence, blocking autophagy using CQ, an autophagic flux blocker, can enhance the apoptotic effect of anticancer drugs [31,32].

**Figure 5:**
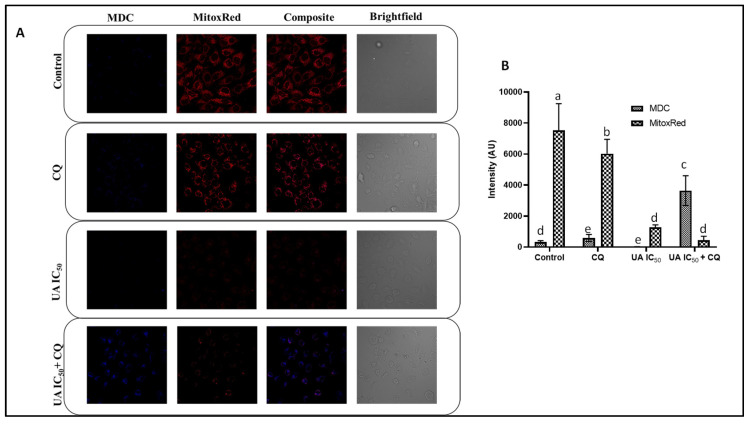
Confocal microscopy of UA-treated HeLa cells. (**A**) Evaluation of autophagy and MMP loss mediated by UA with or without CQ after 24 h of treatment. The cells were dual stained with MDC and MitoxRed and visualized via confocal microscopy. (**B**) Quantification of fluorescence intensities of stained HeLa cells using ImageJ software. Values are the means ± SD of three independent experiments. Means sharing different letters differ significantly from each other at *p* ≤ 0.05.

The cells were treated with UA followed by CQ and visualized via confocal microscopy (Figure 5A,B). The MDC staining of HeLa cells treated with UA IC_50_ + CQ resulted in the accumulation of autophagosomes, as indicated by the presence of blue puncta and elevated blue fluorescence intensity. However, treatment with UA (IC_50_) alone demonstrated the absence of blue fluorescence reflecting the autophagosome degradation via the lysosomal pathway. No blue puncta were observed in control cells and CQ-treated cells. As seen in the histogram (Figure 5B), a massive increase in blue fluorescence intensity was recorded when cells were co-treated with UA and CQ [31]. MitoxRed staining indicated a massive MMP disruption in response to UA (IC_50_) + CQ in comparison to that with UA (IC_50_) in HeLa cells. As seen in the histogram (Figure 5B), compared to that in the control and CQ-treated cells, the red fluorescence decreased drastically when treated with UA and further when co-treated with CQ. This causes severe energy disruptions in the cells and further elevates apoptosis as compared to that with UA alone treatment. UA-induced autophagy has also been observed in HePG2 and SNU-449 (HBV(+) hepatocellular carcinoma (HCC) cell lines [19,20]. Further, the HeLa cells were treated with the phagophore fusion inhibitor chloroquine (CQ) for the inhibition of autophagy. Autophagy inactivation has been associated with decreased JNK activity and increased UA-induced cytotoxicity in HePG2 cells [19]. Mitochondrial dysfunction is also linked to apoptosis and autophagy. Since, autophagy is a pro-survival mechanism, it protects mitochondrial function. However, blocking autophagy elevates the apoptotic effect of anticancer drugs and also induces MMP loss [31]. The interplay between apoptosis and autophagy decides the final fate for the turnover or the destruction of the cellular structures, including cancer cells [33].

### 2.5. Inhibition of Autophagy Increases the Cytotoxicity of UA against HeLa Cells

**Figure 6:**
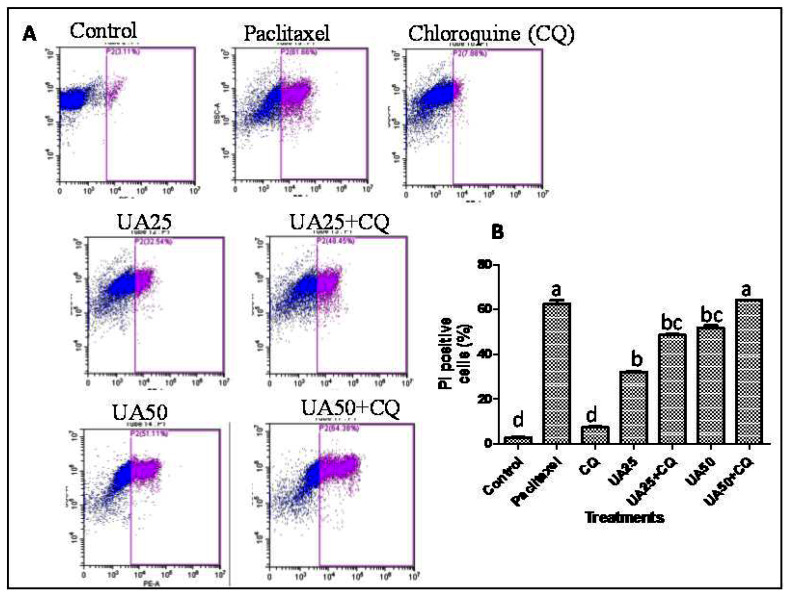
Cytotoxic effect of usnic acid (UA) against HeLa cells in the presence of chloroquine (+/−CQ) via PI live/dead FACS analysis after 24 h of treatment. (**A**) FACS plot of UA-treated (+/−CQ) HeLa cells. (**B**) Bar graph representing PI-positive cells after UA treatment (+/−CQ). Values are the means ± SD of three independent experiments. Means sharing different letters differ significantly from each other at *p* ≤ 0.05. The blur color represents PI negative cells, whereas purple color represents PI positive cells.

After confirming the autophagy-inducing activities of UA and its inhibition mediated by CQ, the role of UA-induced autophagy in cytotoxicity against HeLa cells was investigated. The MTT and resazurin assays were carried out with the UA-treated HeLa cells in the presence and absence of CQ. A significant increase in cell death was observed after the inhibition of autophagy mediated by CQ treatment (Figure 4B,C). The IC_50_ value decreased from 48.65 to 29.1 µM based on the MTT assay carried out on UA-treated cells with CQ treatment, while a 17% decrease in the IC_50_ value of UA was observed in UA-treated HeLa cells after autophagy inhibition during the resazurin assay. The data were cross-verified via PI live/dead flow cytometric assays. An increase of 25–49% cell death was observed after autophagy inhibition in UA-treated HeLa cells (Figure 6A,B). UA is known to induce autophagy, but the regulation of cytotoxicity mediated by autophagy has not been studied in detail. Yurdacan et al. [20] in their studies observed the role of UA as a therapeutic compound against hepatocellular carcinoma (HCC) cells, though they also emphasized further studies on the death-preventive or promoting roles of UA-induced autophagy in cancer cell lines. Many natural products have shown the apoptosis-suppressing role of autophagy mediated by regulating ROS generation and interfering with signaling pathways [34]. Though the interplay of ROS and autophagy is complex, studies have supported the link between decreased ROS and pre-tumoral autophagy, suggesting the important role of autophagy inhibitors in increased cancer cell death [35].

### 2.6. Autophagy Inhibition Increases ROS Generation and Changes the Membrane Potential of UA-Treated Hela Cells

**Figure 7:**
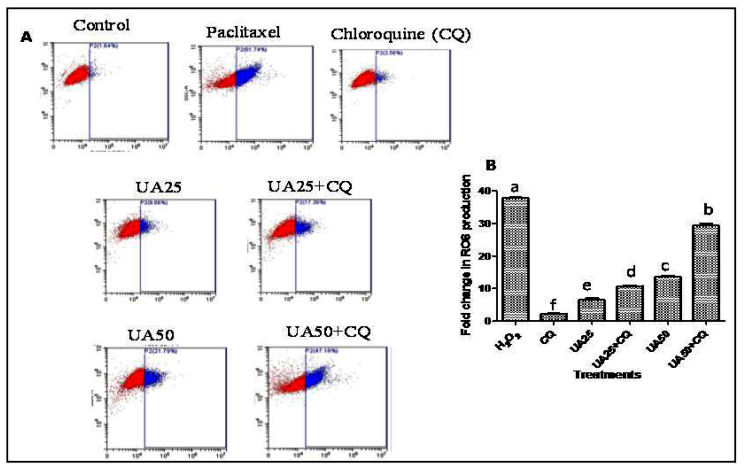
Effect of UA on reactive oxygen species (ROS) production in HeLa cells in the presence of chloroquine (+/−CQ) via DCFHDA FACS analysis after 24 h of treatment. (**A**) FACS plot of UA-treated (+/−CQ) HeLa cells. (**B**) The bar graph shows the fold-change in ROS production after UA treatment (+/−CQ). Values are the means ± SD of three independent experiments. Means sharing different letters differ significantly from each other at *p* ≤ 0.05. The blue color represents ROS generating cells, while red color represents healthy cells which are not producing ROS.

ROS-triggered modulation of redox homeostasis and programmed cell death is a well-known phenomenon in cancer cell death [36]. Modulating the balance between autophagy and redox signaling pathways is the key to the outcome of cancer cells [37]. DCHFDA flow cytometry staining was performed to evaluate the role of autophagy inhibition in ROS generation in UA-treated HeLa cells. It was observed that UA treatment increased the ROS generation in HeLa cells by 6–13-fold. Further, CQ treatment resulted in a significant increase in ROS production in UA-treated HeLa cells (Figure 7A). The ROS production increased 28.75-fold with CQ treatment in comparison to the 13.28-fold increase in 50 µM UA-treated HeLa cells (Figure 7B).

**Figure 8:**
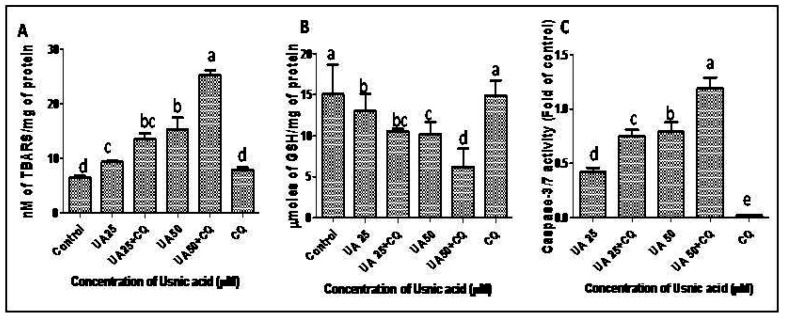
Effect of UA on HeLa cells in the presence or absence of chloroquine after 24 h of treatment; (**A**) increase in lipid peroxidation; (**B**) depletion of reduced glutathione (GSH); (**C**) caspase 3/7 activity. Values are the means ± SD of three independent experiments. Means sharing different letters differ significantly from each other at *p* ≤ 0.05.

Lipid peroxidation and the reduced glutathione level (GSH) in cancer cells are also major determinants of cancer cell fate. GSH depletion and increased lipid peroxidation are hallmarks of cancer cell death [38,39]. After CQ treatment, the lipid peroxidation increased to 44.84–64.66% in comparison to that in UA-treated HeLa cells without CQ treatment (Figure 8A). The GSH level in CQ treated cells was 23.2–40% lower than that in UA-treated HeLa cells without CQ (Figure 8B).

Taken together, the data suggest that autophagy inhibition plays a significant role in increased ROS generation and dysregulation of the redox machinery in UA-treated HeLa cells. The influence of ROS in shaping the cancer microenvironment and the progression of programmed cell death is crucial [40]. The inhibition of autophagy can result in the disruption of redox homeostasis via the accumulation of ROS and upregulation of apoptosis-inducing pathways.

CQ treatment in UA-treated HeLa cells also altered the zeta potential of cells. Zeta potential is generally used for colloidal systems to explore their stability and study the surface charges, though its use in biological systems is untapped [41]. The inhibition of autophagy mediated by CQ resulted in a further decrease in the zeta potential from −23.17 ± 1.33 mV to −29.78 ± 3 in 50 µM UA-treated HeLa cells. The control cells had a zeta potential of −19.08 ± 0.81 (Table S2). A shift in the zeta potential towards the more negative side may indicate the exposure of negatively charged phospholipids, especially phosphatidylserine, to the outer membrane, which occurs during apoptosis. Kazantsev et al. [42] correlated a similar decrease in the zeta potential of neuro-2a tumor cells with cytotoxicity after their exposure to alumina nanoparticles.

### 2.7. Autophagy Inhibition Increases Apoptosis in UA-treated HeLa Cells by Increasing Caspase 3/7 Activity

The caspase 3/7 activity in HeLa cells increased by 0.42- and 0.79-fold after treatment with 25 and 50 µM UA, respectively. The activation of the caspase 3/7 pathway is a pre-apoptotic event that initiates a cascade of reactions for the apoptotic death of cancer cells [43]. The results confirmed the apoptosis-inducing potential of UA in A ROS-dependent manner. Further, the activity of caspase 3/7 was increased by 50–78% in UA-treated HeLa cells when treated with CQ in comparison to that in UA-treated HeLa cells without CQ (Figure 8C). The results indicated that the inhibition of autophagy can increase the cytotoxicity of UA in an apoptosis-dependent manner in HeLa cells. Caspase activation is the ultimate hallmark of apoptosis. Anticancer compounds target autophagy and apoptosis. Although this interplay is extremely complex, several studies have demonstrated the pivotal role of autophagy blockers in enhancing cancer chemotherapy outcomes [44]. Bhattacharjee et al. [45] reported the cytotoxic effect of ormeloxifene in ovarian cancer cells. The cytotoxicity of the compound was characterized by caspase-dependent apoptosis, MMP loss and ROS generation. All of these effects were immensely elevated when co-treatment with an autophagic inhibitor was performed. Since cancer cells can grow in a hypoxic, limited nutrition environment, they are especially capable of utilizing an autophagic survival strategy. Hence, the autophagy–apoptosis concept has been widely researched to suppress autophagy and elevate apoptosis [41,44,46].

## 3. Materials and Methods

### 3.1. Collection and Identification of Lichens

The lichen samples were collected from Kodaikanal (10.2381° N, 77.4892° E), Tamilnadu, India surrounded by Palani Hill Forests and Munnar (10.0889° N, 77.0595° E), Kerela, India, the two locations known for the lichen diversity and richness of fauna (Supporting Figure S1A,B). Samples were collected in paper bags in March, 2019, and transported to the Department of Biochemistry, at the Indian Institute of Science, Bengaluru, under aseptic conditions. The collected lichen thalli were washed with sterile water and air-dried for one week to remove the moisture [12]. The obtained samples were identified using their morphology and the results of a color test using K (5% Ca(OH)_2_), C (aq. Ca(OCl)_2_.), and PD (C_6_H_4_(NH_2_)_2_).

### 3.2. Cell Line and Culture Conditions

The cell lines of human cervical carcinoma (HeLa), human breast cancer (MCF-7) and human lung cancer (A-549) were collected from the collection center of the National Centre for Cell Sciences, Pune, India. The cells were grown in complete DMEM supplemented with 10% FBS (cDMEM), antibiotics (100 mg/L penicillin, 250 mg/L streptomycin) and 2 mM glutamine in a CO_2_ incubator at 37 °C, with 5% CO_2_ and 95% humidity [47].

### 3.3. Preparation of the Lichen Extracts and Screening of the Extracts for Their Cytotoxic Activities in HeLa and MCF Cell Lines

The 10 g of air-dried lichen thalli was ground uniformly to yield a powder form. The dried powder was sequentially extracted with hexane and acetone for non-polar and polar active ingredient extraction. Briefly, the dried powders were soaked in 200 mL of hexane and kept on the rotary shaker for 48 h. Thereafter, the filtrate was again soaked in 200 mL of acetone and kept on the rotary shaker for 48 h. Further, both the non-polar (hexane) and polar (acetone) extracts were obtained by evaporating the organic solvents in a rotary evaporator until the organic solvent was evaporated.

Both the extracts were screened to evaluate their cytotoxic potential using HeLa cells and human breast cancer cells (MCF-7) via MTT assays following standard protocols [48]. In brief, the respective cells were seeded in 96-well plates at a 10^4^ cells/well seeding density and incubated for 24 h in cell culture conditions. Thereafter the cells were exposed to hexane and acetone extracts of lichens (50, 100 and 200 µg/mL) for 24 h., after which the MTT assay was performed by measuring the absorbance at a wavelength of 595 nm on a multiplate reader instrument (Tecan Infinite M200 Pro microplate reader). Paclitaxel (100 nm) served as positive control.

### 3.4. Screening of Selected Extracts for their Cytotoxic Potential in the Presence of Autophagy Inhibitors

The lichen extracts showing cytotoxic potential were further checked for the interference of autophagy [49]. Briefly, the cells were incubated with lichen extracts (25, 50, 100 and 200 µg/mL) for 24 h with 80 µM of chloroquine, an autophagy inhibitor. After the incubation, an MTT assay was carried out as described earlier. Paclitaxel served as positive control. The IC_50_ values with or without treatment with chloroquine were calculated to infer autophagy in the cell death induction mediated by lichen extracts.

### 3.5. Gas Chromatography Mass Spectroscopy (GCMS) Studies for Detection of Cytotoxic Metabolites in Lichen Extracts

To screen the cytotoxic metabolites present in the extracts, GCMS studies were carried out. The lichen extract that showed potential to increase cytotoxicity after autophagy inhibition (UC2A) was subjected to GCMS analysis using the previously described instruments and protocols [47,50]. The compounds spotted in GC/MS were further identified by performing a National Institute of Standards and Technology (NIST) database search.

### 3.6. Purification and Identification of UA from U. cornuta Acetone Extract (UC2A)

#### 3.6.1. Thin Layer Chromatography (TLC)

Based upon the earlier evidence of KC test and GCMS results, TLC of the UC2A extract was carried out using silica gel 60 GF254 TLC sheets (Merck, India), 20 × 20, with a 0.25 mm thickness and the optimized solvent system (toluene:acetic Acid, 9:1). For this, 1 μg/mL of standard + (−) usnic acid was simultaneously run on the same silica gel sheet. Further, the TLC sheet was visualized with UV 254 nm light, and the R_f_ values were noted for each spot. The bands with the same R_f_ value as the standard UA were scrapped, dissolved in acetone, and dried in a speed vacuum for further use.

#### 3.6.2. GCMS and FTIR Analysis

GCMS was used to identify the TLC band and to analyze its mass fragmentation pattern using a mass spectral database search of the NIST, as described earlier. The hit with the highest score was used to elucidate the structure [51].

The functional groups were observed via FTIR analyses (Perkin Elmer, US), and the spectra were observed at a resolution of 4 cm^−1^ in the region of 4000–400 cm^−1^ [51].

### 3.7. Confirmation of Autophagy Inhibition and Change in Mitochondrial Potential using Mitotracker Red (MitoxRed) and Monodansyl Cadaverine (MDC) Staining

MitoxRed and MDC double staining was performed for the control and the UA-treated HeLa cells with or without CQ, which has been used to analyze the formation of acidic vacuoles [32,49]. Briefly, cells at a seeding density of 1 × 10^5^ cells/mL were grown for 24 h and treated with UA (25, 50 µM) with or without CQ. After incubation, the cells were washed thrice and stained with 100 nM MitoxRed and 50 μM MDC at 37 °C in the dark for 30 min. The stained cells were visualized immediately using a confocal laser scanning microscope (Olympus FLUOVIEW FV3000 confocal microscope). The relative intensities of different treatments were plotted further using ImageJ software [52].

### 3.8. In Vitro Cytotoxicity of UA in Human Cancer Cell Lines and Effect of Autophagy Inhibition on the Cytotoxic Effect of UA on HeLa Cells

The cytotoxicity of UA (5–500 µM) isolated and purified from the UC2A extract was checked using HeLa, MCF-7 and A-549 cells after 24 h of incubation by performing MTT assays, as described earlier. Based on the data, IC_50_ values of UA for each cell line were calculated.

The maximum cytotoxicity of UA was obtained with HeLa cells. Therefore, further studies were performed only using HeLa cells. The cytotoxicity of UA (25, 50, 100 µM for 24 h) in the presence of an autophagy inhibitor, chloroquine (80 µM), was checked via MTT assays, as described earlier, and the IC_50_ values were calculated in the presence and absence of chloroquine.

Further, to check the enhancement in UA-induced cell death mediated by the inhibition of autophagy, a resazurin reduction test was performed. Briefly, HeLa cells (1 × 10^4^ cells/mL) were cultured in 96-well plates for 24 h. Thereafter, the cells were treated with UA (25, 50, 100 µM) in the presence and absence of chloroquine (CQ) for 24 h.

After 24 h, 20 μL resazurin (0.01%) was added, and the cells were further incubated at 37 °C in dark. After 3 h, the intensity of the fluorescence was measured using a multiplate reader by focusing the excitation and emission wavelengths at 544 nm and 590 nm, respectively. The cytotoxicity was measured by comparing the UA treated cells with the untreated control, based on which the IC_50_ values were calculated [32].

### 3.9. PI Live/Dead Assay

To assess the changes of in the surface induced by UA, a PI live/Dead assay was performed [22,25]. Before analysis, HeLa cells (3 × 10^5^ cells/mL) were treated with UA (25, 50 µM) with or without CQ for 24 h. The live and dead cells were measured via FACS (Cytoflex, Beckman Coulter) and analyzed using CytExpert 2.0 software [43].

### 3.10. Dichloro-Dihydro-Fluorescein Diacetate (DCFH-DA) Assay

The DCFH-DA assay was used to evaluate the effect of autophagy inhibition on the oxidative stress or generation of reactive oxygen species (ROS) in HeLa cells induced by UA [47]. HeLa cells were administered UA (25, 50 µM) treatment for 24 h either with or without the 80 M CQ treatment. After 15 min of dark incubation at 37 °C, new 10 M DCFH-DA was added to the culture medium, which was then trypsinized, and the cells were harvested. CytExpert 2.0 software (California, USA) was used to quantify and analyze the ROS levels using FACS (Cytoflex). Hela cells were used as the test subjects, while cells treated with 0.1% DMSO served as the positive and negative controls, respectively.

### 3.11. Lipid Peroxidation and Reduced Glutathione (GSH) Assay

To further assess the impact of autophagy on the redox machinery of UA-treated HeLa cells (25, 50 µM) with or without CQ, the extent of lipid peroxidation and depletion of glutathione were assessed [14].

For the lipid peroxidation assay, control and treated HeLa cell lysis was performed with tris-EDTA buffer supplemented with a protease inhibitor cocktail. Further, the TBA-reacting substances (TBARS) were calculated as nanomoles of TBARS/mg protein. The Bradford method followed to measure the protein content of HeLa cells.

To measure the concentration of GSH in lysates of HeLa cells, the cells were treated with 0.1 mM DTNB, 12 mM NADPH and 50 U/L GR. However the generation of 5-thio-2-nitrobenzoic acid (TNB) was measured at 412 nm as n moles/mg of protein.

### 3.12. Surface Potential of the Cells

To check the effect of autophagy inhibition on the cell surface potential in UA-treated cells, the zeta (ξ) potential was measured via dynamic light scattering (ZetaPALS, Brookhaven Instruments, USA) [14,41,47]. Briefly, the UA-treated HeLa cells (25, 50 µM) with or without treatment with CQ were trypsinized, harvested, and suspended in 1 mL of HEPES buffer after 24 h of incubation. The instrument calculated the zeta (ξ) potential based on the electrophoretic mobility of the cells constructed based on the Smoluchowski equation as follows:

v = (εE/η) ξ,

where v = electrophoretic velocity, η = viscosity, ε = electrical permittivity of the electrolytic solution and E shows the electric field.

### 3.13. Caspase 3/7 Assay for Confirmation of Apoptosis in the Presence of an Autophagy Inhibitor

For measuring the caspase 3/7 activation after UA treatment (25, 50 µM) in HeLa cells for 24 h in the presence of an autophagy inhibitor, the Glomax caspase 3/7 assay (Promega, India) was performed [43].

### 3.14. Statistical Analysis

In this study, each experiment was run in triplicate. The quantitative variables were displayed as histograms and expressed as the mean ± S.D using SPSS 16.0 analysis. Statistical significance was considered at a *p*-value ≤ 0.05. [51].

## 4. Conclusions

This study provides insight into the vast cytotoxic potential of lichen extracts isolated from the Western Ghats, India, which can be explored further to hunt for potent compounds from nature’s Pandora box. The study also demonstrated that the inhibition of autophagy in UA-treated HeLa cells increases the cytotoxic potential of UA. The combinatorial application of UA and autophagy inhibitors can be an interesting perspective that needs to be exploited in the search for new cytotoxic agents.

## Figures and Tables

**Table 1:** Cytotoxicity (IC_50_) of lichen acetone and hexane extracts against HeLa and MCF-7 cells after 24 h of treatment. *PS: Parmelia sulcata, UC: Usnea cornuta, PA: Parmelia andinum, PP: Parmotrema perlatum, US: Usnea* sp., *CF: Collema flaccidum, HP: Hypogymnia physodes, CM: Cladonia macilenta, HI: Heterodermia indica, CM: Cladonia macilenta, PS12 :Parmelia* sp. *12, LS: Leptogium* sp., *PS16: Physcia* sp.

S. No	Lichen Extract	Acetone Extract IC_50_ (µg/mL)		Hexane Extract IC_50_ (µg/mL)	
		HeLa	MCF7	HeLa	MCF7
1.	PS1	99.28 ± 3	98.80 ± 8	>200	174.8 ± 6
2.	UC2	38.2 ± 1.25	41.7 ± 2	96.8 ± 11	167.2 ± 7
3.	PA3	79.12 ± 8	93.5 ± 5	>200	170.3 ± 8
4.	PP4	76.87 ± 4	>200	>200	189.3 ± 9
5.	US5	73.2 ± 6	115.02 ± 6	>200	183.09 ± 5
6.	CF6	75.31 ± 1	69.77 ± 9	79.05 ± 8	71.4 ± 8
7.	HP7	75.45 ± 7	71.51 ± 11	191 ± 2	>200
8.	CM8	158.40 ± 13	>200	69.03 ± 7	70.04 ± 4
9.	HI9	188.28 ± 17	>200	>200	>200
10.	CM10	173.03 ± 16	188.28 ± 19	175.7 ± 13	189.1 ± 14
11.	PS11	179.75 ± 11	>200	186.3 ± 16	>200
12.	PS12	115.22 ± 13	>200	86.4 ± 7	82.8 ± 9
13.	CF13	53.76 ± 5	63.8 ± 8	123.1 ± 9	>200
14.	LS14	169.35 ± 9	65.2 ± 2	41.1 ± 3	53.7 ± 5
15.	HP15	89.22 ± 5	>200	59.09 ± 5	58.3 ± 4
16.	PS16	71.88 ± 4	111.06 ± 7	111.9 ± 6	66.2 ± 7

**Table 2:** (A) Cytotoxicity of selected lichen acetone extracts (IC_50_) against HeLa and MCF-7 cells in the presence and absence of the autophagy inhibitor chloroquine (CQ) after 24 h of treatment. (B) Cytotoxicity of selected lichen hexane extracts (IC_50_) against HeLa and MCF-7 cells in the presence and absence of the autophagy inhibitor chloroquine (CQ) after 24 h of treatment.

(A)					
S. No	Lichen Extract	Acetone Extract			
		HeLa		MCF-7	
		−CQ	+CQ	−CQ	+CQ
1.	UC2	38.2 ± 1.25	26.01 ± 1	41.7 ± 2	37.1 ± 2
2.	PA3	79.12 ± 8	78.09 ± 6	93.5 ± 5	92.9 ± 7
3.	CF6	75.31 ± 1	77.56 ± 9	69.77 ± 9	72.7 ± 8
4.	CF13	53.76 ± 5	46.9 ± 6	63.8 ± 8	67.4 ± 9
(B)					
S. No	Lichen Extract	Hexane Extract			
		HeLa		MCF-7	
		−CQ	+CQ	−CQ	+CQ
1.	CF6	79.05 ± 8	72.3 ± 8	71.4 ± 8	66.6 ± 8
2.	CM8	69.03 ± 7	62.8 ± 7	70.04 ± 4	66.9 ± 6
3.	LS14	41.1 ± 3	34.03 ± 1	53.7 ± 5	47.4 ± 4
4.	HP15	59.09 ± 5	58.9 ± 4	58.3 ± 4	59 ± 2

**Table 3:** GCMS analysis of *Usnea cornuta* acetone extract (UC2A) for detection of lichen compounds.

S. No	Name of the Compound	Chemical Formula	Retention Time (Rt)	Area	Relative % Peak Area
1	1,2-Benzenediol, o-(2,3,4-trifluorobenzoyl)-o’-(2,2,3,3,4,4,4-heptafluorobutyryl)-	C_17_H_6_F_10_O_4_	8.758	65,830	0.07
2	Pentasiloxane, dodecamethyl-	C_12_H_36_O_4_Si_5_	8.802	75,562	0.08
3	3,6,9-Trioxa-2,10-disilaundecane, 2,2,10,10-tetramethyl-	C_10_H_26_O_3_Si_2_	10.315	471,048	0.5
4	Hexane, 3,3-dimethyl	C_8_H_18_	10.749	45,591	0.04
5	1,2,3-Trimethyldiaziridine	C_4_H_10_N_2_	10.749	41,170	0.04
6	Glycerol, tris(trimethylsilyl) ether	C_12_H_32_O_3_Si_3_	11.098	1,216,831	1.3
7	3-Penten-2-one, 5,5,5-trimethoxy-, (Z)-	C_8_H_14_O_4_	13.43	25,375	0.02
8	Benzoic acid, 2,4-dihydroxy-3,6-dimethyl-, methyl ester	C_10_H_12_O_4_	14.068	8,253,466	8.8
9	Trimethyl(2,6 ditert.-butylphenoxy)silane	C_17_H_30_OSi	16.362	790,618	0.84
10	Benzoic acid, 2-hydroxy-4-((2-hydroxy-4-methoxy-3,6-dimethylbenzoyl) oxy)-3,6-dimethyl-, methyl ester	C_19_H_20_O_7_	17.18	9,126,518	9.7
11	Pentane, 1-(1-ethoxyethoxy)-	C_9_H_20_O_2_	18.149	13,449	0.01
12	L-(-)-Arabitol, pentakis(trimethylsilyl) ether	C_20_H_52_O_5_Si_5_	19.565	5,036,488	5.3
13	2-Deoxy ribose O,O’,O’’-tris(trimethylsilyl)-	C_14_H_34_O_4_Si_3_	19.72	5,745,306	6.14
14	DL-Glyceraldehyde, tris(trimethylsilyl) ether	C_12_H_30_O_3_Si_3_	19.734	3,791,242	4.05
15	meso-Erythritol, tetrakis(trimethylsilyl) ether	C_16_H_42_O_4_Si_4_	19.964	20,462,416	21.8
16	L-(-)-Arabitol, pentakis(trimethylsilyl) ether	C_20_H_52_O_5_Si_5_	20.12	5,937,654	6.35
17	Adonitol, pentakis(trimethylsilyl) ether	C_20_H_52_O_5_Si_5_	20.174	11,992,669	12.8
18	D-Ribo-Hexitol, 3-deoxy-1,2,4,5,6-pentakis-O-(trimethylsilyl)-	C_21_H_54_O_5_Si_5_	22.039	470,230	0.5
19	Anhydro 5-hydroxy-3-piperonyl-1,2,3,4-oxatriazolium hydroxide	C_9_H_7_N_3_O_4_	22.584	24,373	0.02
20	9,10-Anthracenedione, 2-methyl-1,6-bis[(trimethylsilyl)oxy]-	C_21_H_26_O_4_Si_2_	23.515	240,365	0.25
21	Benzenamine, 2,6-diethyl-	C_10_H_15_N	23.698	39,700	0.04
22	(+)-Usnic acid	C_18_H_16_O_7_	24.358	10,990,722	11.7
23	Hexadecanoic acid, trimethylsilyl ester	C_19_H_40_O_2_Si	24.842	1,843,200	1.97
24	Apovincamine	C_21_H_24_N_2_O_2_	27.26	1,386	0.001
25	Octadecanoic acid, trimethylsilyl ester	C_21_H_44_O_2_Si	27.696	679,631	0.72
26	Hexadecanoic acid, 2,3-bis[(trimethylsilyl)oxy]propyl ester	C_25_H_54_O_4_Si_2_	32.355	740,045	0.79
27	1,4-Benzenedicarboxylic acid, bis(2-ethylhexyl) ester	C_24_H_38_O_4_	34.058	218,519	0.23
28	Octadecanoic acid, 2,3-bis[(trimethylsilyl)oxy]propyl ester	C_27_H_58_O_4_Si_2_	34.609	5,103,911	5.46
29	1-(Bromomethyl)-2,3-bis(4,5-dimethoxy-2-methylphenyl)-2,3-dihydro-4,5-dimethoxy-7-methyl-1H-indene	C_31_H_37_BrO_6_	41.231	11,108	0.011

## Data Availability

The data that support the findings of this study are available from the corresponding author upon reasonable request.

## Supplementary Materials

The following supporting information can be downloaded at: https://www.mdpi.com/article/10.3390/plants12030519/s1, Figure S1: Sampling locations of lichens in Munnar and Kodaikanal, India, Figure S2: Structure of barbatic acid and atraric acid, Table S1: Diversity and distribution pattern of lichens isolated from the Western Ghats, India, Table S2: Zeta potential of usnic acid (UA) treated HeLa cells in the presence and absence of chloroquine (+/−CQ) after 24 h of treatment.
