# Supplementary material for: Inhibition of Autophagy Increases Cell Death in HeLa Cells through Usnic Acid Isolated from Lichens
Source: Plants (Basel). 2023 Jan 23;12(3):519. doi: 10.3390/plants12030519 (PMC9919968; doi:10.3390/plants12030519)
Supplement: Supplementary file 1 [file plants-12-00519-s001.zip › plants-2153973-supplementary.pdf]

Inhibition of Autophagy Increases Cell Death in HeLa Cells through Usnic Acid Isolated from Lichens

Madhuree Kumari [1], Siya Kamat [1], Sandeep Kumar Singh [2], Ajay Kumar [3,*] and C. Jayabaskaran [1,*]

[1] Department of Biochemistry, Indian Institute of Science, Bangalore 560012, India
[2] Division of Microbiology, Indian Agricultural Research Institute, Pusa, New Delhi 110012, India
[3] Centre of Advanced study in Botany, Banaras Hindu University, Varanasi 221005, India
* Correspondence: ajaykumar_bhu@yahoo.com (A.K.); cjb@iisc.ac.in (C.J.);Tel.: +91-80-2293-2482; Fax: +91-80-2360-0814 (C.J.)

A

B

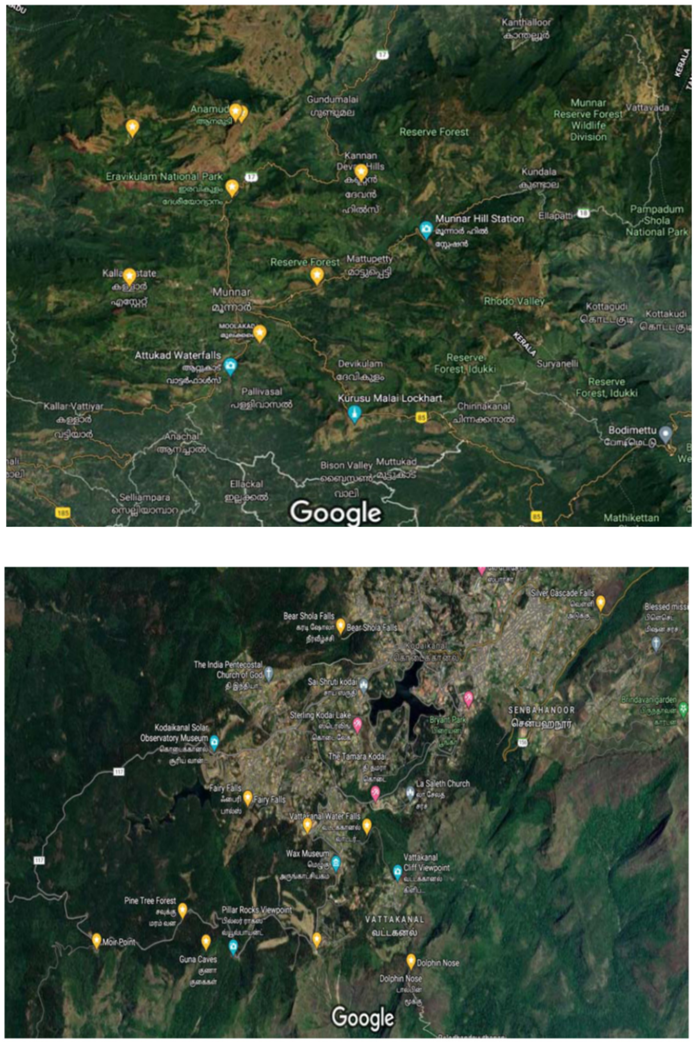

Figure S1: Sampling locations of lichens in Munnar and Kodaikanal, India

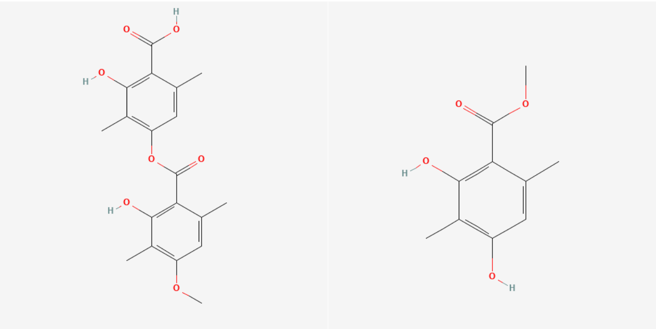

Barbatic acid $C_{19}H_{20}O_7$ Atraric acid $C_{10}H_{12}O_4$

Figure S2: Structure of barbatic acid and atraric acid

Table S1: Diversity and distribution pattern of lichens isolated from the western Ghats, India

S. no.	Lichen name	Location	Description	Spot test
1.	*Parmelia sulcata*	Bear Shola falls, Kodaikanal	Growing on the bark of the tree	KC-P+yellow
2.	*Usnea cornuta*	Bear Shola falls, Kodaikanal	Growing on the bark of the tree	KC+pale yellowP+orange yellow e
3.	*Parmelia andinum*	Guna caves, Kodaikanal	Growing on the bark of the tree	KC-P+orang
4.	*Parmotrema perlatum*	Dolphin nose, Kodaikana	Growing on the bark of the tree	KC-P-
5.	*Usnea sp.*	Dolphin nose, Kodaikana	Growing on the bark of the tree	KC+pale yellowP+orange
6.	*Collema flaccidum*	Pine tree/Moir point, Kodaikanal	Growing on the bark of the tree	KC-P-
7.	*Hypogymnia physodes*	Pine tree/Moir point, Kodaikanal	Growing on the bark of the tree	KC-P+yellow
8.	*Cladonia macilenta*	Dolphin nose, Kodaikana, Kodaikanal	Growing on the bark of the tree	KC+P+yellow
9.	*Heterodermia indica*	Bear Shola falls, Kodaikanal	Growing on the rocks	KC-P+yellow

10.	*Cladonia macilenta*	Anamudi, Munnar	Growing on the bark of the tree	KC+P+ yellow
11.	*Parmelia sulcata*	Kannan devan Hills	Growing on the tree in tea garden	KC-P+yellow
12.	*Parmelia sp.*	Kannan devan Hills, Munnar	Growing on the tree in tea garden	KC-P+yellow
13.	*Collema flaccidum*	Kallar estate, Munnar	Growing on the tree in tea garden	KC-P-
14.	*Leptogium sp.*	Eravikulam National Park, Munnar	Growing on the tree in tea garden	KC-P-
15.	*Hypogymnia physodes*	Eravikulam National Park, Munnar	Growing on the bark of the tree	KC-P+yellow
16.	*Physcia sp.*	Anamudi, Munnar	Rocks	KC-P-

Table S2: Zeta potential of usnic acid (UA) treated HeLa cells in the presence and absence of chloroquine (+/-CQ) after 24h of treatment

**UA (µM)**	**Zeta potential (mV)**
Control	-19.08±0.81
25	-20.01±1.08
25+CQ	-23.15±0.35
50	-23.17±1.33
50+CQ	-29.78±3
C+CQ	-20.45±0.66
UA (1mM)	8.05±0.98
CQ	-6.02±1.2
